# Increasing the diagnostic yield of childhood glaucoma cases recruited into the 100,000 Genomes Project

**DOI:** 10.1186/s12864-024-10353-8

**Published:** 2024-05-16

**Authors:** Omayma Al-Saei, Samantha Malka, Nicholas Owen, Elbay Aliyev, Fazulur Rehaman Vempalli, Paulina Ocieczek, Bashayer Al-Khathlan, Genomics England Research Consortium, Khalid Fakhro, Mariya Moosajee

**Affiliations:** 1 Institute of Ophthalmology, University College London, London, EC1V 9EL UK; 2 Department of Human Genetics, Sidra Medicine, PO Box 26999, Doha, Qatar; 3 Moorfields Eye Hospital NHS Foundation Trust, London, EC1V 2PD UK; 4 Genomic Data Science Core, Sidra Medicine, PO Box 26999, Doha, Qatar; 5 The Francis Crick Institute, London, NW1 1AT UK

**Keywords:** Childhood glaucoma, Congenital glaucoma, Genetic eye disorders, Developmental eye diseases, Whole genome sequencing, Genomics

## Abstract

Childhood glaucoma (CG) encompasses a heterogeneous group of genetic eye disorders that is responsible for approximately 5% of childhood blindness worldwide. Understanding the molecular aetiology is key to improving diagnosis, prognosis and unlocking the potential for optimising clinical management. In this study, we investigated 86 CG cases from 78 unrelated families of diverse ethnic backgrounds, recruited into the Genomics England 100,000 Genomes Project (GE100KGP) rare disease cohort, to improve the genetic diagnostic yield. Using the Genomics England/Genomic Medicine Centres (GE/GMC) diagnostic pipeline, 13 unrelated families were solved (13/78, 17%). Further interrogation using an expanded gene panel yielded a molecular diagnosis in 7 more unrelated families (7/78, 9%). This analysis effectively raises the total number of solved CG families in the GE100KGP to 26% (20/78 families). Twenty-five percent (5/20) of the solved families had primary congenital glaucoma (PCG), while 75% (15/20) had secondary CG; 53% of this group had non-acquired ocular anomalies (including iris hypoplasia, megalocornea, ectopia pupillae, retinal dystrophy, and refractive errors) and 47% had non-acquired systemic diseases such as cardiac abnormalities, hearing impairment, and developmental delay. *CYP1B1* was the most frequently implicated gene, accounting for 55% (11/20) of the solved families. We identified two novel likely pathogenic variants in the *TEK* gene, in addition to one novel pathogenic copy number variant (CNV) in *FOXC1*. Variants that passed undetected in the GE100KGP diagnostic pipeline were likely due to limitations of the tiering process, the use of smaller gene panels during analysis, and the prioritisation of coding SNVs and indels over larger structural variants, CNVs, and non-coding variants.

**Supplementary Information:**

The online version contains supplementary material available at 10.1186/s12864-024-10353-8.

## Background

Childhood glaucoma (CG) comprises a group of rare diseases that manifests before 18 years of age and is typically more severe than adult glaucoma [1]. According to the Childhood Glaucoma Research Network (CGRN), CG is classified as primary or secondary [2]. Primary childhood glaucomas include primary congenital glaucoma (PCG) and juvenile open-angle glaucoma (JOAG), whereases secondary childhood glaucomas include those associated with non-acquired ocular anomalies (such as Axenfeld-Rieger spectrum (ARS), aniridia, and Peters anomaly), glaucoma associated with non-acquired systemic diseases (such as connective tissue disorders including Marfan syndrome, Weill-Marchesani syndrome, and Stickler syndrome), and glaucoma associated with acquired conditions (such as trauma, infections, or surgeries) [2]. Glaucoma post cataract surgery has a different classification [2]. Childhood glaucoma commonly presents as bilateral disease with features of photophobia, epiphora and blepharospasm [3, 4].

Children with PCG develop buphthalmos if disease onset is before 3 years of age secondary to the raised intraocular pressure (IOP), increased corneal diameter (> 12 mm), Haab striae, corneal oedema, optic disc cupping and progressive myopia [3, 5, 6]. Aqueous fluid secreted by ciliary body is recycled in the anterior segment of the eye via the porous trabecular meshwork (TM) [7]. Defects in the TM and the iridocorneal angle of the anterior chamber, caused by maldevelopment of neural crest tissues, can hinder the drainage process leading to fluid accumulation in the anterior chamber with resultant raised IOP [8]. Gonioscopy can reveal that the angle has an immature appearance of arrested development with a high flat iris insertion, with peripheral scalloping and circumferential iris vessels [9]. Elevated IOP can consequently cause loss of retinal ganglion cells (RGCs) and a progressive optic neuropathy [4, 10–12], however optic disc cupping can be reversible with treatment [13]. Globally, the incidence rate of PCG is approximately 1–80 cases per 100,000 live births [10, 14]. This prevalence varies geographically and may rise by 5 to 10 times [15], in highly consanguineous populations such as in Slovakian Roma (1/1250) [16] and Saudi Arabia (1/2766) [17]. It is one of the most significant causes of childhood blindness worldwide [4, 15].

Prognosis and management of CG relies principally on prompt, precise diagnosis, and effective control of the IOP and prevention of amblyopia to preserve visual function [18–20]. Medical management with both topical and oral drugs can be used as a temporary modality or as an adjunct to surgery, but surgery remains the predominant treatment in order to control the IOP [3, 19]. However, periodic examinations and lifelong follow-ups are essential, as congenital glaucoma can worsen with complications impairing the visual function later in life [21, 22].

The genetic aetiology of CG is not fully understood, however, it is often associated with variants in genes exhibiting Mendelian inheritance [23, 24]. For PCG, the most prevalent genes implicated are *CYP1B1*, *LTBP2*, and *TEK* [23], while genes such as *MYOC*, *TBK1*, and *OPTN* are mainly associated with JOAG. Variants in genes *FOXC1*, *PITX2*, *PAX6*, and *CPAMD8* contribute to CG associated with non-acquired ocular anomalies [7, 23]. High penetrance with variable expressivity are common, resulting in phenotypic heterogeneity and overlapping clinical features [25]. Only 10–40% of the PCG cases are familial with a history of consanguinity, the majority of cases are sporadic [26].

The *CYP1B1* (cytochrome P450, family 1, subfamily B, polypeptide 1) gene, located on chromosome 2p21-p22 [27], is well studied and is the most predominately linked gene to autosomal recessive PCG [24]. More than 200 *CYP1B1* variants have been linked to PCG [28], accounting for approximately 87% of the familial cases and 27% of sporadic cases [29]. The expression of *CYP1B1* has been detected in various human tissues including the heart, brain, skeletal muscles [30], and several ocular tissues such as the iris, ciliary body, cornea, retinal epithelium [31, 32], but its expression in the TM remains controversial [31, 33]. *CYP1B1* has also been implicated in both vitamin A metabolism and transcription induction of genes necessary for the proliferation and differentiation of multiple ocular elements [34, 35]. In *Cyp1b1*^*-/-*^ mice, anomalies in the architecture of TM and Schlemm’s canal (SC) of mice eyes were reported, resembling the ocular features of human PCG [36]. Further, various reports indicated ocular hypertension in *Cyp1b1*^*-/-*^ mice induced by increased levels of oxidative stress, corresponding to the changes seen in human glaucomatous TM tissues, suggesting the role of *CYP1B1* in the suppression of oxidative stress [37].

The myocilin gene, *MYOC*, is expressed in the sclera, TM, ciliary body, retina, myocardium, and other non-ocular tissues [38–40]. *MYOC* has been associated with juvenile and primary open-angle glaucoma (POAG) [41, 42] accounting for 2–5% of the POAG cases [43, 44], and accounts for 5.5% of the PCG cases [45]. It has been suggested that defects in the MYOC protein affect the structure of TM and ciliary body, blocking the drainage of fluid, and rising IOP [46, 47]. Besides, the accumulation of mutant myocilin in TM cells leads to endoplasmic reticulum (ER) stress and the activation of the unfolded protein response (UPR) cascade [48]. Interestingly, a possible functional interaction between *CYP1B1* and *MYOC* has been identified, in which the former acts as a modifier [49]. Causative variants in *CYP1B1* negatively impact its ability to metabolise 17β estradiol resulting in the overexpression of *MYOC* and can potentially lead to the development of glaucoma [49].

The forkhead box protein C1 (*FOXC1*) gene is expressed in human adult iris, foetal craniofacial tissues, and other non-ocular tissues [50], and its murine homologue is abundantly expressed in the periocular mesenchyme and anterior segment tissues during eye development [51]. Variants in *FOXC1* are associated with Axenfeld-Rieger syndrome/anomaly [50, 52], Peters anomaly [53], PCG [1, 52], and increased susceptibility to POAG [54]. A recent study reported that out of 131 PCG patients, 8 (6.1%) harboured pathogenic *FOXC1* variants [1]. Additionally, extra-ocular features related to Axenfeld-Rieger syndrome (such as hearing impairment, cardiac abnormalities, and developmental delay) are frequently present in human PCG patients harbouring *FOXC1* variants [1]. Mice with *Foxc1* variants showed defects in the development of the anterior segment structures, comparable to the clinical ocular features of human patients [55].

The *LTBP2* gene, encoding the extracellular matrix (ECM) latent transforming growth factor (TGF)-β binding protein 2, mapped to 14q24.3 [56], has also been implicated in PCG [57–59]. Variants in *LTBP2* have been identified in PCG families from Pakistan, Iran, and in Slovakian Roma [7]. Additionally, recessive variants in this gene were detected in patients with megalocornea, lens dislocation, spherophakia, secondary glaucoma, and Marfan-like syndrome [60–62]. *LTBP2* is expressed in the heart, placenta, skeletal muscle, liver [56], as well as in the ocular anterior segment, TM, and the ciliary body, thus may have a role in the morphogenesis of the anterior chamber and maintenance of its muscular structure [57, 58]. Furthermore, *LTBP2* knockdown in human TM cell cultures parallels the effects of oxidative stress induction, and both influence the expression of ECM genes and apoptosis in the TM cells, which may be mediated by the activation of the canonical TGF-β and BMP signaling pathways [63].

Approximately 5% of the PCG cases have been associated with haploinsufficiency of the angiopoietin receptor *TEK* gene [64, 65]. *TEK*-related PCG families have been reported to exhibit autosomal dominant inheritance with incomplete penetrance and phenotypic variation [64]. Aside from its expression in the developing embryonic vascular system in mice [66] and in human endothelial and haematopoietic cells [67, 68], it is also expressed in the SC [69, 70]. Deletion of *Tek* gene in mice led to the progressive degeneration of the SC, severe ocular hypertension, deterioration of retinal ganglion cells, and glaucoma [71]. It has been suggested that a functioning *TEK* gene is crucial during the normal development of the iridocorneal angle in the anterior chamber of the eye [64].

The ground-breaking efforts of the Genomics England 100,000 Genomes Project (GE100KGP) have resulted in the sequencing of more than 100,000 whole genomes of more than 88,000 participants, of which 82% of cases were enrolled into the Rare Disease cohort, since the inception of the project in 2013 through 2018 [72]. Participants were recruited through hospitals linked to one of 13 NHS genomic medicine centers across England [72]. The GE100KGP created platforms and automated processing pipelines for variant calling, quality check, and interpretation [72]. All participants underwent de-identification, and their clinical and genomic data are stored in a secure setting within the Genomics England (GE) research environment [72]. Academic researchers can access these data through a membership in one of the GE clinical interpretation partnership domains or through approved collaborations [72]. In the GE pilot study, WGS data of 4660 participants from more than 2000 families were analysed, and 25% of these cases received definitive diagnosis [73]. Out of these diagnoses, 14% were made in genomic regions that conventional genetic testing would have missed [73]. Beyond the immediate diagnosis, these data are also of significance to researchers who continue to use them to inform new diagnoses, and to develop effective therapies and drugs [73].

Despite insights into the molecular aetiology of CG, no gene-directed therapies are available as yet and the majority of patients lack a genetic diagnosis. Current diagnostic rates of CG and anterior segment dysgenesis sit between 24.5% and 33.9% using targeted gene panels, whole exome/genome sequencing, or mixed testing approaches [25, 74, 75]. To further improve the diagnostic yield of CG, we conducted an *in silico* analysis by leveraging genome sequencing data of unsolved (or partially solved) CG cases from the GE100KGP [72] for variants in genes of interest. Using the CGRN classification, our cohort included primary and secondary CG associated with non-acquired ocular anomalies or non-acquired systemic diseases. Glaucomas caused by acquired conditions such as ocular surgeries, traumas, or tumours were excluded.

## **Methods**

### Patient cohort

Using the LabKey software within the GE research environment, Rare Disease participants who had been recruited into the GE100KGP between 2013 and 2018 for WGS were queried. Unsolved patients who were recruited for Developmental glaucoma (HP:0001087) as a primary diagnosis in addition to those with the HPO terms Primary congenital glaucoma (HP:0008007), Late onset congenital glaucoma (HP:0008041), and Buphthalmos (HP:0000557) were included in this analysis.

### Whole genome sequencing

Whole genome sequencing was performed through the GE100KGP as described previously [76]. Briefly, TruSeq DNA PCR-Free Sample Preparation kit (Illumina Inc.) was used to extract genomic DNA. Whole genome sequencing (WGS) was done using high-throughput HiSeq X Ten platform (Illumina Inc.), yielding a coverage of 15X for the majority (> 97%) of the callable autosomal genome. Alignment of reads was done via Isaac (Illumina Inc.) using the reference human genome (assemblies GRCh37 or GRCh38). Variant calling was done using Starling (v2.4.7, Illumina Inc.) for single nucleotide variants (SNVs) and indels (insertion or deletions), Canvas [77] for copy number variants (CNVs), and Manta [78] for structural variants (SVs).

### In silico analysis

Genes of interest were mainly selected based on the traffic light system of the PanelApp [79], where only the 18 Green (diagnostic-grade) genes of the glaucoma (developmental) panel (version 1.42) were prioritised during interpretation, as they reflect high level of evidence for genotype-phenotype associations. Whereas Amber and Red genes were generally excluded as they represent borderline and low level of evidence, respectively. Twenty-two additional genes from other PanelApp panels (Structural eye disease (version 3.2), Corneal abnormalities (version 1.12), or Cataracts (version 4.1)) or identified in CG cohorts reported in the literature were also included in our analysis [80, 81, 90–98, 82–89] (Table S1). Coordinates of the coding regions of our gene panel (40 glaucoma-associated genes) were retrieved using Ensembl BioMart [99, 100] and variant call files (VCF) with SNVs, insertions and deletions (indels), and SVs were scanned over these regions and filtered. Annotation of extracted variants was done using Ensembl Variant Effect Predictor (VEP; v99) [101] for SNVs and indels, and AnnotSV (v3.1.1) [102] for CNVs and SVs. Using stepwise filtering, annotated SNVs were prioritised according to minor allele frequency (< 0.01) in the GE100KGP database and population databases (such as gnomAD (v.4.1.0) [103] and TopMed [104]), impact (high or moderate), consequence type, mode of inheritance (in familial cases), location within the gene, and deleteriousness (as predicted by *in silico* tools such as CADD [105], SIFT [106], Polyphen-2 [107], MutationTaster2 [108]). Other databases such as HGMD [28] (public version), ClinVar [109], and VarSome (v11.10) [110], were examined along with literature searches for published reports of variants identified in this cohort. Variants classified as pathogenic or likely pathogenic according to ACMG criteria were prioritised. For the SVs, only the duplications and/or deletions that overlapped exonic regions of the genes of interest with ACMG scores > 3 were prioritised. Confirming the presence of variants was manually performed using Integrative Genomics Viewer (IGV) and Samplot (for large duplications and deletions). Genetic findings were evaluated by an interdisciplinary team (including bioinformaticians, clinical scientists, and specialists in ophthalmic genetics) to validate variant deleteriousness. Novel variants detected in this study were submitted to ClinVar.

### Further phenotyping

The medical records of the Moorfields Eye Hospital (MEH) glaucoma patients recruited into the GE100KGP were inspected for visual acuity, refraction, initial and recent IOP, glaucoma surgeries, additional ocular phenotypes, systemic features, and family history. Fundus photographs were also examined, wherever possible.

## **R**esults

### Genomics England (GE100KGP) cohort

Using the Human Phenotype Ontology (HPO) terminologies in LabKey, we identified a list of 86 unique participants from 78 unrelated families with CG. Fifteen cases from 13 unrelated families were solved using the Genomics England/Genomic Medicine Centres (GE/GMC) diagnostic pipeline [111]. The whole genome sequencing data of the remaining 71 cases, from 65 unrelated families, were further interrogated using an expanded gene panel (40 genes reported to be associated with glaucoma), by investigating SNVs as well as structural and copy number variations in these genes. The CG cohort identified has a mean age of 21.0 ± 14.1 years (ranges from 6 to 76 years), of which 64% (55/86) were male patients. The majority of the patients were White British (63%; 54/86), followed by Asian Pakistani (9%; 8/86), White Irish (8%; 7/86), White Other (6%; 5/86), Asian Other (5%; 4/86), Black Caribbean (5%; 4/86), Black British (3%; 3/86), and Black Other (1%; 1/86) (Figure S1, Table S2).

#### Ocular and systemic features

**Fig. 1:**
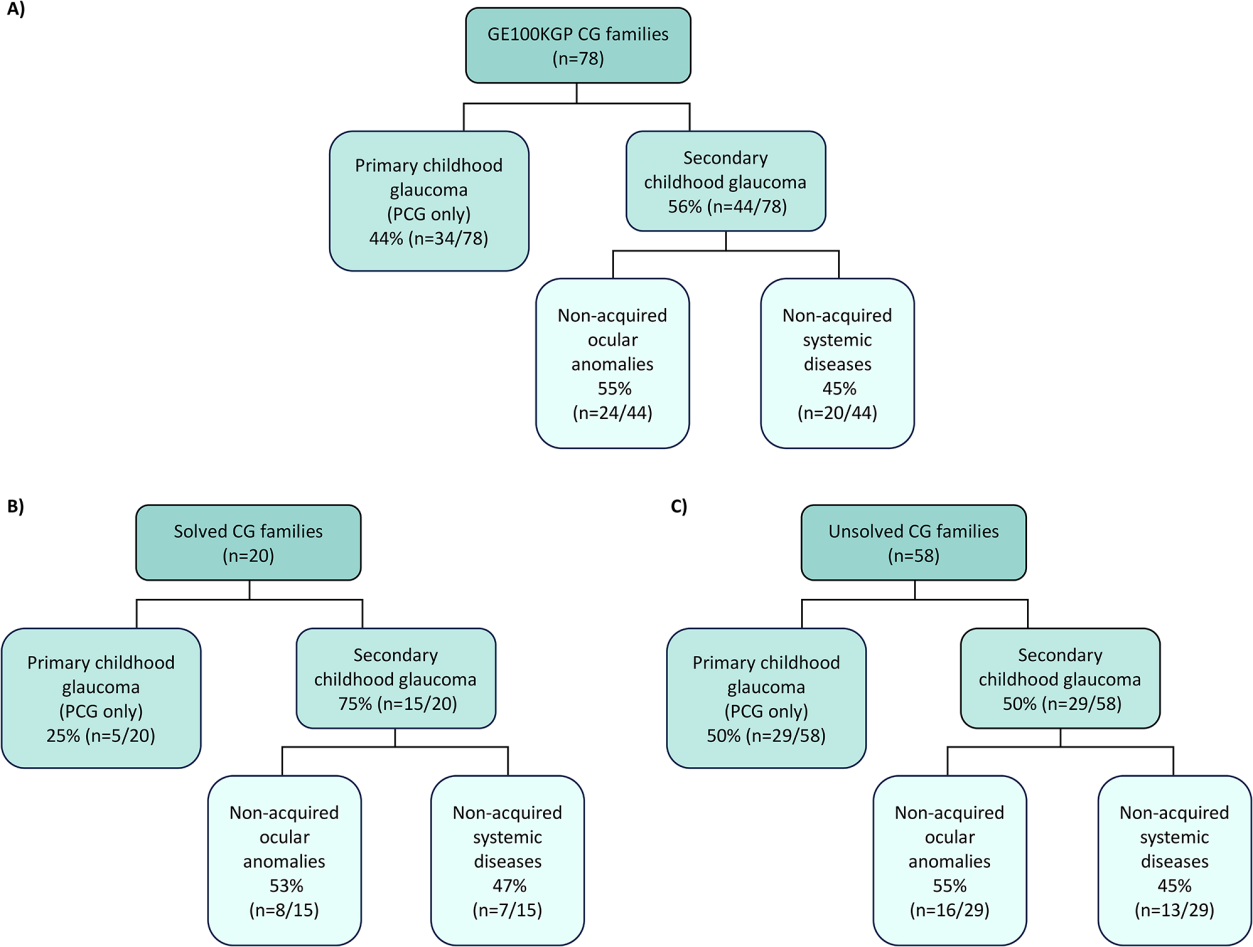
**Classification of clinical features in the childhood glaucoma (CG) cohort of GE100KGP**. **(A)** Overview of the glaucoma classification in the whole CG cohort. (**B)** Classification of CG features in the solved families of the CG cohort. (**C)** Classification of CG in the remaining unsolved cases of the CG cohort

Of the patient cohort, 44% (34/78) of the families had PCG, whereas 56% (44/78) had secondary glaucoma (Fig. 1A). Of the secondary glaucoma group, 55% (24/44) exhibited non-acquired ocular anomalies including 58% (14/24) with corneal abnormalities, 29% (7/24) with iris anomalies (including iris hypoplasia, ectopia pupillae, and polycoria), 29% (7/24) with cataracts, 25% (6/24) with anterior segment dysgenesis (ASD), 17% (4/24) had retinal disorders, 13% (3/24) had refractive error, and 8% (2/24) displayed nystagmus (Table S2). Given the limited clinical data available in the GE research environment, it could not be confirmed if the cataracts were diagnosed prior to glaucoma or post glaucoma surgery (except for case GEL-064-01; Table 1). 45% (20/44) of the secondary glaucoma cohort exhibited non-acquired systemic features including 30% with deformities of the spine or extremities (6/20), 15% with growth disorders (3/20), 15% with hearing impairment (3/20), 10% with cardiovascular abnormalities (2/20), 5% had a collagenopathy (1/20), 5% had cancer (1/20), and 40% had multisystem anomalies encompassing defects in more than three systems (8/20) (Table S2).

**Table 1:** Clinical and genetic features of probands identified with potential pathogenic variants in the solved GE100KGE CG cohort

Family ID	Patient ID	Gender		Age (Years)	Ethnicity	Gene	Clinical diagnosis (MIM number)	Primary CG/ secondary CG	NAOA/NASD	Ocular phenotype(s)	Systemic features
GEL-002*	GEL-002-01		F	9	Asian Pakistani	*CYP1B1*	Congenital glaucoma (231300)	Primary	.	CG	Facial dysmorphism including down slanted palpebral fissures; ear abnormality; 1–2 toe syndactyly; microcephaly; cerebellar atrophy; developmental delay
GEL-007	GEL-007-01		F	9	Asian Pakistani	*SLC4A11*	CHED (217700)	Secondary	NAOA	CG; corneal oedema	.
	GEL-007-04		F	7				Secondary	NASD	CG; corneal oedema; unilateral corectopic pupil	Cardiac murmur
GEL-033	GEL-033-01		F	27	White British	*TEK*	Congenital glaucoma (617272)	Secondary	NAOA	CG; megalocornea	.
GEL-048	GEL-048-01		M	10	White British	*FOXC1*	Axenfeld-Rieger syndrome, type 3 (602482)	Secondary	NASD	CG	Primary ciliary dyskinesia; situs inversus totalis; dextrocardia; bilateral conductive hearing impairment
GEL-050	GEL-050-01		F	24	White British	*TEK*	Congenital glaucoma (617272)	Secondary	NAOA	CG; megalocornea; iris hypoplasia	.
GEL-056	GEL-056-01		F	11	Asian Other	*CYP1B1*	Congenital glaucoma (231300)	Primary	.	CG	.
GEL-064	GEL-064-01		M	27	White British	*SBF2*	Charcot-Marie-Tooth disease type 4B2 (604563)	Secondary	NASD	CG; cataracts (post surgery)	CMT; severe autism; depression; chronic fatigue syndrome; demyinating poluneuropathy; pes cavus; hydromyelia
GEL-S01	GEL-S01-01		F	20	Black Caribbean	*SOS2*	Noonan syndrome 9 (616559)	Secondary	NASD	CG; ptosis	Noonan syndrome; developmental delay; cardiovascular abnormalities; hearing abnormality; peripheral neuropathy; lymphedema
GEL-S02	GEL-S02-01		M	30	Asian Other	*FOXC1*	ASD 3 (601631)	Primary	.	CG	.
GEL-S03	GEL-S03-01		F	22	White British	*CYP1B1*	Congenital glaucoma (231300)	Primary	.	CG	.
GEL-S04	GEL-S04-01		M	26	White British	*CYP1B1*	Congenital glaucoma (231300); ASD 6 (617315)	Secondary	NAOA	CG; ectopia pupillae (RE); cataracts (LE)	.
GEL-S05	GEL-S05-01		M	24	White Other	*CYP1B1*	ASD 6 (617315)	Secondary	NAOA	CG; ASD; cataracts	.
	GEL-S05-04		F	8				Secondary	NAOA	CG; ASD; corneal oedema; iris and chorioretinal coloboma (LE)	.
GEL-S06	GEL-S06-01		M	19	White British	*CYP1B1*	Congenital glaucoma (231300); ASD 6 (617315)	Secondary	NAOA	CG; nystagmus; cataracts; ASD	.
GEL-S07	GEL-S07-01		M	22	White British	*CYP1B1*	Congenital glaucoma (231300)	Primary	.	CG	.
GEL-S08	GEL-S08-01		M	33	Asian Other	*COL18A1*	Knobloch syndrome, type 1, AR (267750)	Secondary	NASD	CG; high myopia; retinal dystrophy- part of Knobloch	Collagenopathy
GEL-S09^#^	GEL-S09-01		M	18	White British	*FOXC1*	Axenfeld-Rieger syndrome, type 3 (602482)	Secondary	NASD	CG; myopia; Haab striae; optic disc cupping	Flat feet; abnormal shoulder positioning
GEL-S10	GEL-S10-01		M	36	Asian Pakistani	*CYP1B1*	Congenital glaucoma (231300); ASD 6 (617315)	Secondary	NAOA	CG (unilateral; RE); ASD; cataracts	.
GEL-S11	GEL-S11-01		M	25	Black Caribbean	*CYP1B1*	Congenital glaucoma (231300); ASD 6 (617315)	Secondary	NAOA	CG; cataracts; nystagmus; ASD; astigmatism; myopia	.
	GEL-S11-04		M	21				Primary	.	CG	.
GEL-S12^$^	GEL-S12-01		F	19	White British	*CYP1B1*	Congenital glaucoma (231300); ASD 6 (617315)	Secondary	NASD	GC; iris hypoplasia; ASD	Sensorineural hearing impairment
GEL-S13	GEL-S13-01		M	20	Asian Other	*CYP1B1*	Congenital glaucoma (231300)	Primary	.	CG	.

**Summary of each patient harbouring variant(s) identified in the regions of interest**. Electronic and written notes of Moorfields Eye Hospital patients were examined, where available. AD: autosomal dominant; AR: autosomal recessive; ARS: Axenfeld-Rieger syndrome; ASD: anterior segment dysgenesis; CG: childhood glaucoma; CHED: congenital hereditary endothelial dystrophy of cornea; CMT: Charcot Marie tooth; F: female; LE: left eye; M: male; RE: right eye. *GEL-002 is diagnosed with PCG and their systemic abnormalities are considered due to a secondary unrelated genetic cause. ^#^Family GEL-S09 was previously reported in Jackson et al. (2020) (Family 25,760) [74]. ^$^Hearing impairment in GEL-S12 is likely caused by other genetic factors

#### Molecular diagnosis

**Fig. 2:**
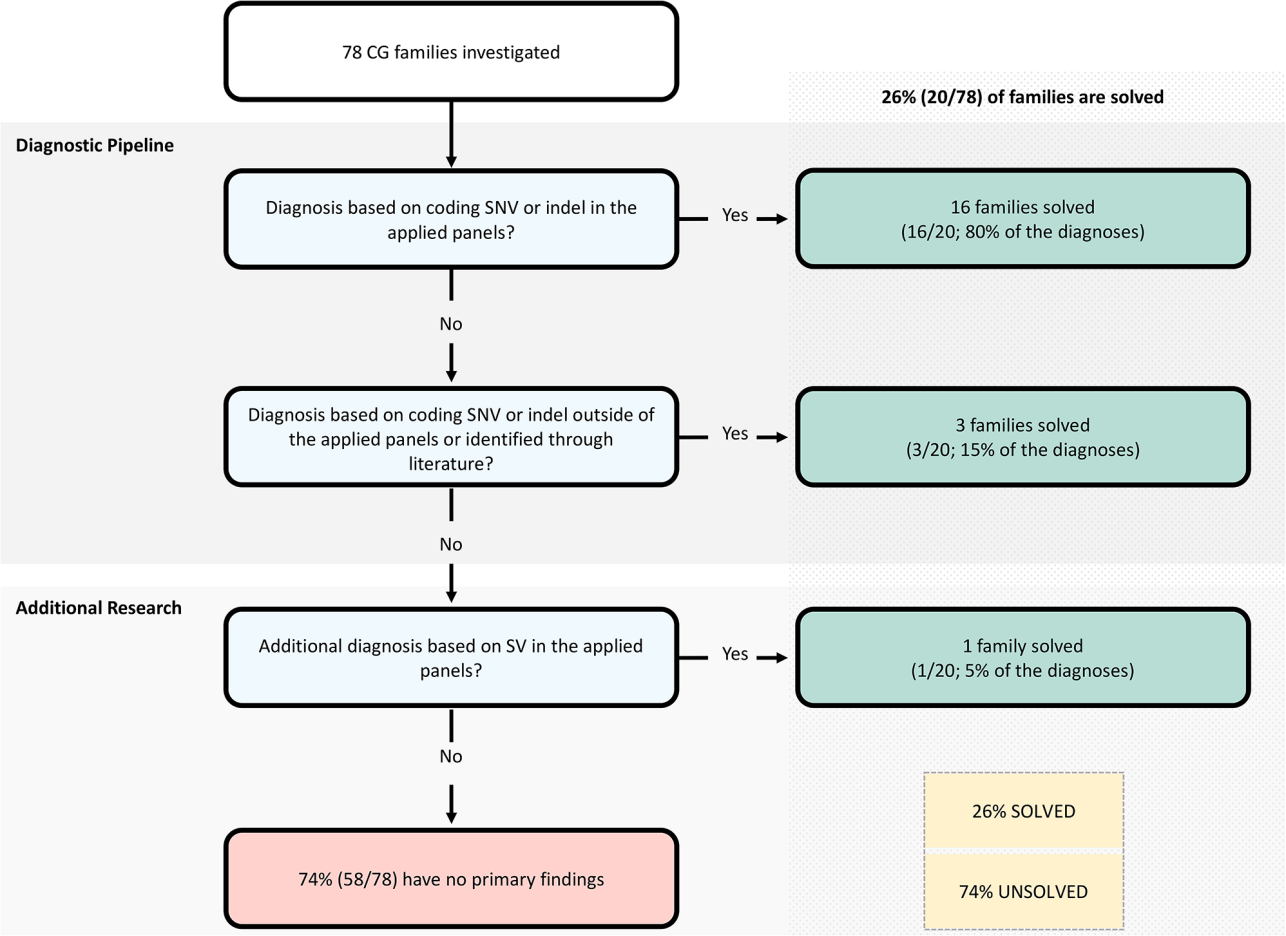
**The diagnostic and research pipeline used in this analysis.** Out of the 20 solved families, 16 were solved based on coding single nucleotide variants (SNVs) or insertions/deletions (indels) in genes from the developmental glaucoma panel, 3 were solved based on coding SNVs or indels outside the developmental glaucoma panel, whereas 1 were solved based on structural variants in genes from the developmental glaucoma panel. Flowchart adapted from Fig. 1 in “100,000 Genomes Pilot on Rare-Disease Diagnosis in Health Care - Preliminary Report”, by Smedley, et al., 2021, *N Engl J Med*, 385(20), p. 1873

A genetic diagnosis was confirmed in 23 CG cases from 20 unrelated families (26%; 20/78) (Fig. 1B; Table 1, and Table S3). Recessive causative variants were homozygous in 50% (10/20) of the affected families and compound heterozygous in 20% (4/20) of the families, while 30% (6/20) of the families inherited variants in an autosomal dominant manner (Table S3). The majority of families (74%; 58/78) remain unsolved with no clear primary findings (Fig. 1C). Approximately 80% (16/20) of the solved families obtained a molecular diagnosis based on coding SNVs or indels in genes determined by the glaucoma panel in PanelApp, followed by 15% (3/20) who received a diagnosis based on SNVs or indels outside the PanelApp, in genes identified through literature search. The remaining 5% (1/20) were diagnosed based on SVs in an applied gene panel (Fig. 2).

Of the solved families, 25% (5/20) had PCG, while the remaining 75% (15/20) had secondary glaucoma, with 53% (8/15) being glaucoma associated with non-acquired ocular anomalies including iris anomalies, cataracts, ASD, retinal detachment, band keratopathy, refractive error, nystagmus, iris and chorioretinal coloboma, and optic nerve anomalies, and 47% (7/15) being associated with non-acquired systemic features including facial dysmorphism, microcephaly, cerebellar atrophy, hearing loss, dextrocardia, ciliary dyskinesia, collagenopathy, Charcot-Marie-Tooth (CMT) disease, autism spectrum disorders, in addition to intellectual and developmental delays (Table 1; Fig. 1B).

**Fig. 3:**
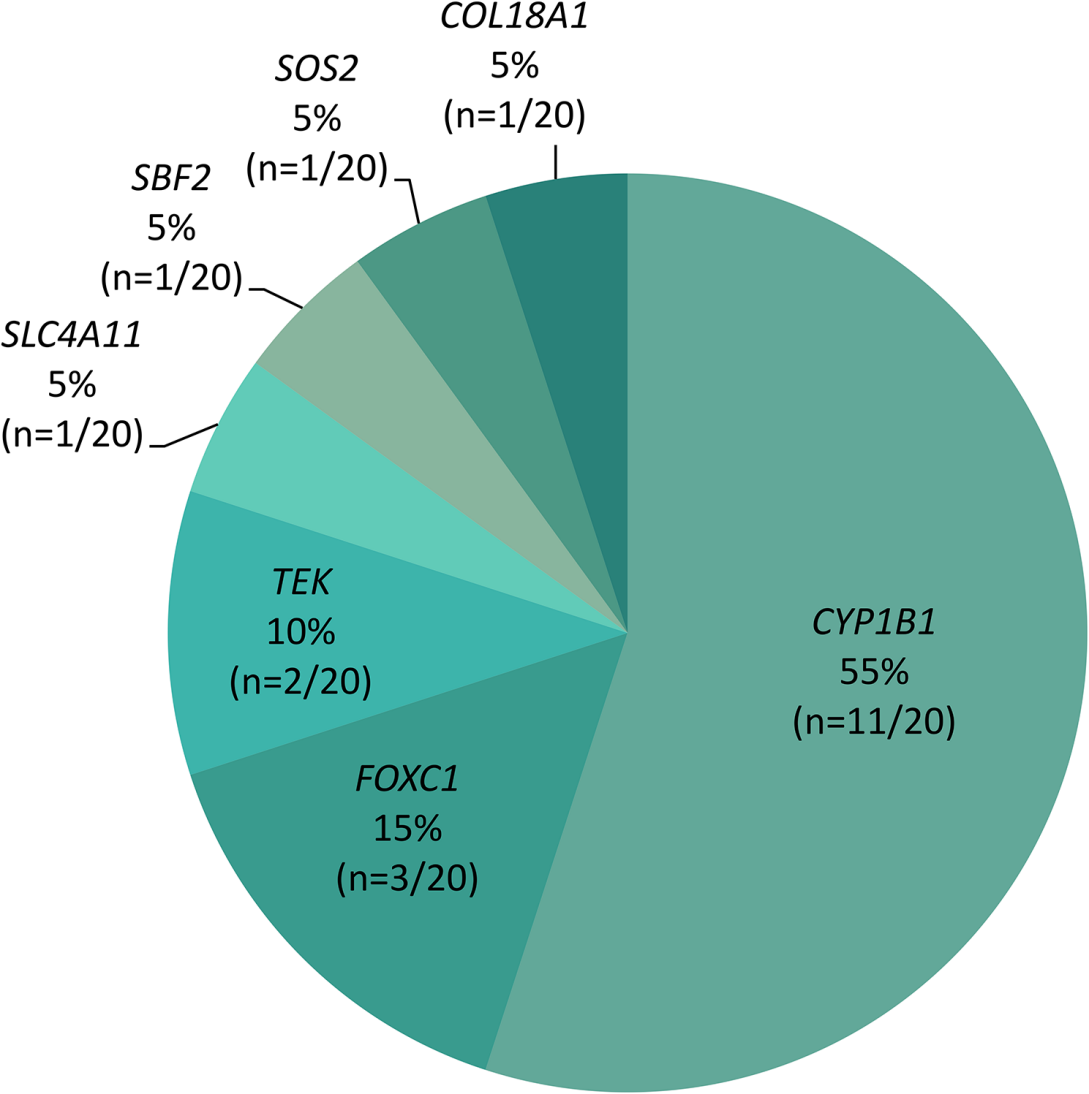
**Monogenic pathogenic causes identified in the solved CG families.** The mutational spectrum of families who received molecular diagnosis in this study. Majority (55%) of the families had variants in *CYP1B1* gene, followed by 15% with variants in *FOXC1*, 10% in *TEK*, and 5% in *SLC4A11*, *SBF2*, *SOS2*, and *COL18A1* (1 family each)

Our search strategy identified 22 potential disease-causing variants mapping to 7 genes including 55% (12/22) in *CYP1B1* (11 families), 14% (3/22) in *FOXC1* (3 families), 9% (2/22) in *TEK* (2 families), 9% (2/22) in *SBF2* (1 family), and 5% (1/22) in *SLC4A11*, *COL18A1*, and *SOS2* (1 family each) (Fig. 3). All variants were either SNVs or indels, except for the novel 2.3Kb deletion in *FOXC1* ((NC_000006.12:g.(1610150_1612452)del); overlapping exon 1). Two novel SNVs were identified to be likely associated with the phenotype, both of which were in the *TEK* gene, *TEK*(NM_000459.5):c.3011G>A p.(Trp1004*) and *TEK*(NM_000459.5):c.475+1G>T (Table 2). Only two (9%; 2/22) non-coding splice site variants (*TEK*(NM_000459.5):c.475+1G>T and *SBF2*(NM_030962.4):c.(2536+1G>A)) were detected in this cohort.

**Table 2:** ACMG/AMP classification of the pathogenic or likely pathogenic single nucleotide variants identified

Gene	cDNA / GRCh38	Protein	PVS1	PS3	PM1	PM2	PM5	PP3	PP5	Class.	Family ID
*COL18A1*	NM_001379500.1 c.3523_3524del 21:45510090:CCT:C	p.(Leu1175Valfs*72)	**Y**	.	.	< 0.001 in gnomAD	.	.	**Y**	P	GEL-S08
*CYP1B1*	NM_000104.4 c.171G>A 2:38075218:C:T	p.(Trp57*)	**Y**	.	.	< 0.001 in gnomAD	.	.	**Y**	P	GEL-S06*
*CYP1B1*	NM_000104.4 c.182G>A 2:38075207:C:T	p.(Gly61Glu)	.	.	**Y**	< 0.001 in gnomAD	**Y**	**Y**	**Y**	P	GEL-002; GEL-S11*
*CYP1B1*	NM_000104.4 c.840C>A 2:38074549:G:T	p.(Cys280*)	**Y**	.	.	< 0.0001 in gnomAD	.	.	**Y**	P	GEL-S07*
*CYP1B1*	NM_000104.4 c.868dup 2:38074520:C:CG	p.(Arg290Profs*37)	**Y**	.	.	< 0.001 in gnomAD	.	.	**Y**	P	GEL-056; GEL-S13
*CYP1B1*	NM_000104.4 c.1064_1076del 2:38071277:TTCTGCCTGCACTC:T	p.(Arg355Hisfs*69)	**Y**	.	.	< 0.001 in gnomAD	.	.	**Y**	P	GEL-S04*
*CYP1B1*	NM_000104.4 c.1139A>G 2:38071215:T:C	p.(Tyr380Cys)	.	.	**Y**	< 0.00001 in gnomAD exomes	.	**Y**	**Y**	LP	GEL-S11*
*CYP1B1*	NM_000104.4 c.1147G>A 2:38071207:C:T	p.(Ala383Thr)	.	.	**Y**	< 0.00001 in gnomAD exomes	.	**Y**	**Y**	LP	GEL-S06*
*CYP1B1*	NM_000104.4 c.1159G>A 2:38071195:C:T	p.(Glu387Lys)	.	.	**Y**	< 0.001 in gnomAD	.	**Y**	**Y**	P	GEL-S04*; GEL-S05
*CYP1B1*	NM_000104.4 c.1168 C>T 2:38071186:G:A	p.(Arg390Cys)	.	.	**Y**	< 0.0001 in gnomAD	**Y**	**Y**	**Y**	P	GEL-S07*
*CYP1B1*	NM_000104.4 c.1169G>A 2:38071185:C:T	p.(Arg390His)	.	.	**Y**	< 0.0001 in gnomAD	**Y**	**Y**	**Y**	P	GEL-S10
*CYP1B1*	NM_000104.4 c.1333T>A 2:38071021:A:T	p.(Phe445Ile)	.	.	**Y**	< 0.00001 in gnomAD exomes	**Y**	**Y**	**Y**	P	GEL-S03
*CYP1B1*	NM_000104.4 c.1345del 2:38071008:TC:T	p.(Asp449Metfs*8)	**Y**	.	.	< 0.0001 in gnomAD	.	.	**Y**	P	GEL-S12
*FOXC1*	NM_001453.3 c.235 C>A 6:1610680:C:A	p.(Pro79Thr)	.	**Y**	**Y**	Absent from controls	**Y**	**Y**	**Y**	P	GEL-S02
*FOXC1*	NM_001453.3 c.1009_1012dup 6:1611451:G:GCGGC	p.(Ala338Glyfs*191)	**Y**	.	.	Absent from controls	.	.	**Y**	P	GEL-S09
*SBF2*	NM_030962.4 c.620G>T 11:10002689:C:A	p.(Gly207Val)	.	.	.	Absent from controls	.	**Y**	**Y**	P	GEL-064*
*SBF2*	NM_030962.4 c.2536+1G>A 11:9853539:C:T	.	**Y**	.	.	Absent from controls	.	.	**Y**	P	
*SLC4A11*	NM_001174089.2 c.1343G>A 20:3230587:C:T	p.(Gly448Asp)	.	.	.	< 0.000001 in gnomAD exomes	.	**Y**	**Y**	LP	GEL-007
*SOS2*	NM_006939.4 c.800T>A 14:50182521:A:T	p.(Met267Lys)	.	**Y**	**Y**	Absent from controls	**Y**	**Y**	**Y**	P	GEL-S01
*TEK*	NM_000459.5 c.475+1G>T 9:27168606:G:T	.	**Y**	.	.	Absent from controls	.	**Y**	.	LP	GEL-050
*TEK*	NM_000459.5 c.3011G>A 9:27217707:G:A	p.(Trp1004*)	**Y**	.	.	Absent from controls	.	.	.	LP	GEL-033

**ACMG/AMP criteria for the classification of the identified variants**. PVS1 is related to frameshift, nonsense, or splice site variants; PS3 is related to functional studies supporting the damaging effect of gene or variant; PM1 is related to missense variants in hotspot regions or functional domains; PM2 is related to the absence or presence at low frequencies in population databases like gnomAD, ExAC, 100 K, 1000 genomes; PM5 refers to alternative variants that have been determined to be pathogenic; PP3 related to computational predictions of pathogenicity; PP5 relates to pathogenic variants reported by reputable sources. Class: classification; LP: likely pathogenic; P: pathogenic; Y: Yes. *Variants in these families were inherited in compound heterozygous fashion, with variant phase being determined from parental data

According to the classification guidelines of the American College of Medical Genetics and Genomics and the Association for Molecular Pathology (ACMG/AMP), 16 of the SNVs and indels were classified as pathogenic (P) and 5 were classified as likely pathogenic (LP) (Table 2). The copy number variant (CNV) in *FOXC1* scored class 5 according to the ACMG criteria, which is considered pathogenic. All variants detected were in genes previously associated with glaucoma and are “Green” in the glaucoma panel of PanelApp except *SLC4A11* and *COL18A1* which are not in the glaucoma panel, but are rated “Green” in the corneal abnormalities (v1.12) and structural eye disease (v3.2) panels, respectively. However, the involvement of *SLC4A11* and *COL18A1* with glaucoma cases has been reported in the literature [88, 112]. Furthermore, *SOS2* is rated “Green” in the foetal anomalies panel (3.133), but recent studies have showed a probable association between variations in *SOS2* and glaucoma [113, 114].

#### Genotype-phenotype correlations

##### *CYP1B1*

Variants in *CYP1B1* (MIM number: 601771), associated with PCG and juvenile- or adult-onset primary open angle glaucoma (MIM: 231,300), were identified in 55% (11/20) of the solved families, and were either homozygous or compound heterozygous. Out of the 11 families, 55% (6/11) had missense variants, 27% (3/11) had frameshift variants, while 18% (2/11) had both nonsense and missense variants (Table 2 and Table S3). One of these families (GEL-S11) was multiplex and exhibited phenotypic heterogeneity amongst affected siblings, in which one individual had PCG and the other had secondary glaucoma associated with non-acquired ocular anomalies including ASD, cataracts, nystagmus, myopia, and astigmatism. Of the remaining 10 families, 5 exhibited PCG, whereas the remaining 5 exhibited secondary glaucoma- 4 had non-acquired ocular anomalies including ectopia pupillae, iris hypoplasia, iris and chorioretinal coloboma, ASD, cataracts, and nystagmus, whereas 1 had glaucoma associated with non-acquired systemic diseases including hearing impairment (Table 2 and Table S3).

##### *FOXC1*

The forkhead box c1 gene (*FOXC1*; MIM number: 601090), associated with ASD (MIM: 601631) and ARS (MIM: 602482) was implicated in 3 families, who harboured heterozygous variants in *FOXC1*. Family GEL-S02 and GEL-S09 had a missense (c.235C>A; p.Pro79Thr) and frameshift (c.1009_1012dup p.(Ala338Glyfs*191)) variant, respectively. Only GEL-S02 had PCG, whereas GEL-S09 had ARS associated with secondary GC, myopia, and skeletal abnormalities. Additionally, family GEL-048 had a structural variant (NC_000006.12:g.(1610150_1612452)del), which was associated with secondary glaucoma and non-acquired systemic complications including primary ciliary dyskinesia, dextrocardia, and conductive hearing impairment (Table 2 and Table S3).

##### *TEK*

The tyrosine kinase gene (*TEK*; MIM: 600221) associated with autosomal dominant PCG, was identified in families GEL-033 and GEL-050. Each had a novel variant in *TEK*; one was a nonsense variant (c.3011G>A p.(Trp1004*)) and the other was a splice site variant (c.475+1G>T), respectively. Both families exhibited secondary glaucoma with non-acquired ocular anomalies including megalocornea and iris hypoplasia (Table 2 and Table S3).

##### *SLC4A11*

The *SLC4A11* gene (MIM: 610206), associated with autosomal recessive congenital hereditary endothelial dystrophy (CHED; MIM: 217700), was detected in the multiplex family GEL-007, two affected individuals had a homozygous missense variant (c.1343G>A p.(Gly448Asp)). The proband (GEL-007-01) had secondary glaucoma with corneal oedema, while her sister (GEL-007-04) had additional corectopic pupil and non-acquired systemic features (cardiac murmur) (Table 2 and Table S3).

##### *SBF2*

The set-binding factor 2 gene (*SBF2*; MIM: 607697), associated with Charcot-Marie-Tooth (CMT) disease, type 4B2 (MIM: 604563) was detected in the proband of family GEL-064 who harboured biallelic compound heterozygous variants; a missense variant (c.620G>T p.(Gly207Val)) and a splice site variant (c.2536+1G>A). The proband (GEL-064-01) exhibited secondary CG with non-acquired systemic features characterised by CMT, severe autism, and depression (Table 2 and Table S3).

##### *SOS2*

*SOS2* gene (MIM: 601247) associated with Noonan syndrome 9 (MIM: 616559) was found in family GEL-S01 with one affected individual (GEL-S01-01) with a heterozygous missense variant (c.800T>A p.(Met267Lys)). The proband had secondary CG with non-acquired systemic disorders characterised by Noonan syndrome associated with cardiovascular anomalies, hearing abnormality, intellectual disability, and developmental delay (Table 2 and Table S3).

##### *COL18A1*

The collagen, type xviii, alpha-1 gene (*COL18A1*; MIM: 120328) linked to autosomal recessive Knobloch syndrome (KS) type 1 (MIM: 267750) was identified in the proband of family GEL-S08. He has a homozygous frameshift variant in *COL18A1* (c.3523_3524del p.(Leu1175Valfs*72)) associated with secondary CG with non-acquired systemic disorders including collagenopathy, in addition to ocular manifestations such as high myopia and retinal dystrophy (Table 2 and Table S3).

#### Variants of unknown significance

Eight unrelated patients were found to have 8 heterozygous variants of uncertain significance (VUS) in *ADAMTS17* (NM_139057.4), *CPAMD8* (NM_015692.5), *CYP1B1* (NM_000104.4), *GJA1* (NM_000165.5), *MYOC* (NM_000261.2), *TEK* (NM_000459.5), *THBS1* (NM_003246.4), and *WDR36* (NM_139281.3) (Table 3 and Table S4). The variant in *ADAMTS17* was a 6.2Kb duplication (NC_000015.10:g.(100146719_100152954)dup) of unknown significance. Current evidence is insufficient to prove disease causality of these variants, therefore, further investigation is required to confirm the pathogenicity.

**Table 3:** ACMG/AMP classification of the single nucleotide variants of uncertain significance identified

Gene	cDNA / GRCh38	Protein	OMIM	PVS1	PS3	PM1	PM2	PM5	PP2	PP3	PP5	BS2	BP1	BP4	BP6	Class.
*CPAMD8*	NM_015692.5 c.4157C>A 19:16904320:G:T	p.(Thr1386Asn)	AR	.	.	.	< 0.0001 in gnomAD	.	.	**Y**	.	.	**Y**	.	.	VUS*
*CYP1B1*	NM_000104.4 c.1103G>A 2:38071251:C:T	p.(Arg368His)	AR	.	**Y**	**Y**	< 0.01 in gnomAD	**Y**	.	.	**Y**	.	.	**Y**	**Y**	VUS*
*GJA1*	NM_000165.5 c.962G>T 6:121447809:G:T	p.(Gly321Val)	AD, AR	.	.	.	< 0.0001 in gnomAD	.	**Y**	.	.	.	.	.	.	VUS
*MYOC*	NM_000261.2 c.526del 1:171652085:TC:T	p.(Glu176Argfs*2)	AD	Y	.	.	< 0.00003 in gnomAD exomes	.	.	.	.	**Y**	.	.	.	VUS
*TEK*	NM_000459.5 c.691G>C 9:27172678:G:C	p.(Gly231Arg)	AD	.	.	.	Absent from controls	.	.	**Y**	.	.	.	.	.	VUS
*THBS1*	NM_003246.4 c.2571 C>G 15:39592606:C:G	p.(Asp857Glu)	N/A	.	.	.	Absent from controls	.	.	**Y**	.	.	.	.	.	VUS
*WDR36*	NM_139281.3 c.2060 C>T 5:111121053:C:T	p.(Ser687Leu)	N/A	.	.	.	< 0.0001 in gnomAD	.	.	**Y**	.	.	.	.	.	VUS

**ACMG/AMP criteria for the classification of variants of uncertain significance (VUS) identified in the cohort**. PVS1 is related to frameshift, nonsense, or splice site variants; PS3 is related to functional studies supporting the damaging effect of gene or variant; PM1 is related to missense variants in hotspot regions or functional domains; PM2 is related to the absence or presence at low frequencies in population databases like gnomAD, ExAC, 100K, 1000 Genomes; PM5 refers to alternative variants that have been determined to be pathogenic; PP2 is related to a gene where missense variation is a common cause of disease; PP3 is related to computational predictions of pathogenicity; PP5 relates to pathogenic variants reported by reputable sources. BS2 is related to variants observed in healthy adult populations; BP1 is related to missense variants identified in a gene where truncating variation is a common cause of disease; BP4 refers to multiple in silico algorithms suggesting no effect on gene or gene product; BP6 refers to a reliable source reporting the variant as benign. Class: classification; LP: likely pathogenic; P: pathogenic; VUS: variant of uncertain significance; Y: Yes. *These variants had conflicting evidence of pathogenicity in the literature, so were considered VUS in this analysis

### Further phenotyping (Moorfields Eye Hospital patients)

Within the GE100KGP CG cohort, there were a total of 48 Moorfields Eye Hospital (MEH) CG patients from 41 unrelated families who underwent WGS, of which 14 cases from 10 unrelated families were solved in this analysis with pathogenic variants in *CYP1B1*, *COL18A1*, *FOXC1*, *SBF2*, *SLC4A11*, and *SOS2* (Table 1). Three of these are consanguineous families (GEL-007, GEL-S05, and GEL-S11). Additionally, 8 families (11 cases) had glaucoma-related surgeries (ranging from goniotomy, trabeculectomy, aqueous shunt implantation, and ciliary body cyclophotocoagulation), with an improvement in IOP reported in all individuals. Only case GEL-064-01 was reported to have developed bilateral cataracts post glaucoma surgery (at 9 years of age), and corneal decompensation was observed in GEL-S05-01 post surgery. Of this MEH series, 1 family had PCG (GEL-S07), while the remaining 9 families had secondary CG, of which 4 had non-acquired ocular anomalies such as corneal oedema, retinal dystrophy, cataracts, and refractive errors, and 5 families exhibited non-acquired systemic features ranging from a cardiac murmur (GEL-007-04), collagenopathy (GEL-S08-01), skeletal anomalies (GEL-S09-01), hearing impairment (GEL-S12-01) and multiple morbidities (GEL-064-01) including Charcot-Marie-Tooth disease, demyelinating polyneuropathy, hydromyelia, autism spectrum disorder, anxiety, depression, and chronic fatigue (Table 1, Tables S2 and S3).

## **Discussion**

Childhood glaucoma is a developmental eye disorder associated with significant genetic and phenotypic variability. A substantial proportion of childhood blindness is attributed to this severe, progressive glaucoma, necessitating early detection and management. Besides monogenic factors, the aetiology of CG is further complicated by interactions of gene regulatory networks, resulting in it being associated with additional non-acquired ocular features and/or systemic manifestations as seen in 56% (44/78) of our patient cohort. Herein, we report a comprehensive characterisation of the GE100KGP’s CG cohort, with an extensive description of the genetic and phenotypic spectrum in 78 CG families undergoing WGS. This work also highlights the crucial role of multidisciplinary care with detailed evaluation of patient phenotype and medical history to improve clinical diagnosis and inform genetic counselling.

The diagnostic yield for the CG cohort in the GE100KGP (including the solved families by the GE/GMC diagnostic pipeline) is now 26% (20/78), which is comparable to the diagnostic rate of CG recently reported in an Australasian cohort (30.4%) [25]. The current diagnostic rate of the GE/GMC diagnostic pipeline for rare diseases is 20.3% [115]. Variants identified in this analysis were previously missed by the GE/GMC diagnostic pipeline for a variety of plausible reasons including the variant tiering process, which omitted many population-specific pathogenic variants (that exceeded an allele frequency of 0.01 for autosomal recessive and 0.001 for autosomal dominant variants in one subpopulation), filtering methodologies, eliminating disease-causing non-coding variants [116], the use of smaller gene panels that lacked some of the recently discovered disease-causing genes at the time of analysis, in addition to the prioritisation of small variants (SNVs/Indels) analysis and dismissal of larger (structural and copy number) variants [115, 116]. The residual majority of CG families remain unsolved (74%; 58/78), which may partially be explained by the limited sample size, the presence of variants in non-coding regions which exert regulatory roles on gene expression but are more challenging to interpret and typically require experimental validation [117]. Analysis is limited to pre-defined gene panels linked to specific phenotypes/diseases, and thus runs the risk of overlooking diagnoses and genes beyond the those in the panels applied. In a recent study, the DeNovoLOEUF tool was used to filter for rare, *de novo*, loss-of-function (LoF) variants in disease-causing genes in all rare diseases trios (13,949) in the GE100KGP [118]. Out of the 332 variants detected, 324 (98%) were diagnostic or partially diagnostic, and 39 diagnoses were identified, which were overlooked by the typical analysis of the GE100KGP data [118]. Applying such tool could potentially solve further cases in the CG cohort.

Approximately 90% (18/20) of the solved families obtained a molecular diagnosis based on variants (SNVs or CNVs) in genes determined by the PanelApp (Fig. 2). Our understanding of the genotype-phenotype correlations is constantly evolving. Therefore, this illustrates the necessity of global partnerships to improve virtual gene panel curation and address inconsistencies, which can ultimately enable healthcare systems to incorporate and participate in data exchange [119].

*CYP1B1*, was the most prevalent gene in the GE100KGP CG cohort, accounting for a total of 13 cases from 11 unrelated families (11/20, 55%). In a cohort with mostly Caucasian participants [73], this prevalence (1 in 7) of *CYP1B1* is comparable to that reported in literature (1 in 5) [47, 120–122]. The majority of *CYP1B1*-related families (55%) had secondary CG, while 45% of them had PCG. Interestingly, the proband GEL-002-01 had PCG consistent with the homozygous inheritance of the pathogenic variant (c.182G>A p.(Gly61Glu)) in *CYP1B1*. However, she also exhibited significant multisystem disorders, and after discussion within a regional multidisciplinary genetics team, it was concluded that there was likely to be another unrelated genetic cause for these systemic features. The precise function of *CYP1B1* in the human eye and CG development is still unknown. In mouse models, however, the deficiency of *Cyp1b1* appears to be involved in maldevelopment of the anterior eye structures, which regulate the aqueous humour outflow pathway [36, 123, 124]. Since glaucoma eventually results from defects in the RGCs due to increased sensitivity to IOP changes, the influence of *Cyp1b1* on the development of RGCs under normal and stressed conditions, such as elevated ocular pressure, was investigated [123]. It was found that where deletion of *Cyp1b1* alone is insufficent to demonstrate glaucomatous features in mice, it may increase the susciptibility of RGCs to degeneration in reponse to elevated IOP [123].

Two patients in the cohort (GEL-033-01 and GEL-050-01) had heterozygous variants in the *TEK* gene, associated with secondary glaucoma and megalocornea in both cases. The first patient had a loss-of-function variant *TEK*(NM_000459.5):c.3011G>A p.(Trp1004*), while the other had a novel splice donor variant *TEK*(NM_000459.5):c.475+1G>T, which was also carried by her asymptomatic mother. The splice donor variant occurring at the boundary of exon 3, is predicted by the SpliceAI [125] and the Human Splicing Finder [126] to affect splicing, and is classified as likely pathogenic according to the ACMG guidelines (Table 2). The proper translation of proteins is highly dependent on the pre-mRNA splicing machinery [127]. Alterations to sequences at the splicing regions could disrupt the splicing system, resulting in exon skipping, new cryptic exons, or the development of new exon/intron junctions, that would consequently affect the processing of transcripts [127, 128], and may affect the production or function of proteins, which could explain the phenotype observed in the patient. However, molecular and functional validations are required to investigate the precise impact of the splicing variant on the function of the *TEK* gene and help establish genotype-phenotype correlations. Additionally, previous studies have reported asymptomatic carriers without the classic early-onset CG phenotype in family members of affected individuals, suggesting that *TEK* gene may be associated with variable penetrance and expressivity in terms of severity and age of onset [64, 65], in keeping with this case.

In our cohort, 3 families (GEL-048, GEL-S02, and GEL-S09) harboured heterozygous autosomal dominant variants in *FOXC1*, one of which had a clinical diagnosis of PCG (GEL-S02), while the remaining 2 cases had ARS associated with CG, hearing impairment, and congenital heart defects (in GEL-048), and glaucoma with myopia and skeletal anomalies characterised by flat feet and abnormal shoulder positioning (in GEL-S09). It has been established that variations in *FOXC1* gene are often associated with a wide range of abnormalities such as various types of glaucoma and systemic anomalies, including hearing loss [129]. This emphasizes the need for testing the *FOXC1* gene in cases of CG particularly, as clinical symptoms of ARS can be subtle and go undetected [1].

Case GEL-S01-01 was diagnosed with Noonan syndrome associated with intellectual disability, cardiac abnormalities, hearing impairment, and ocular manifestations including CG and ptosis. The patient was found to have a missense variant in *SOS2*, a gene known to be associated with Noonan syndrome [130]. Furthermore, *SOS2* was found to be expressed in the human TM [113], and recent genome-wide and whole-exome studies have identified risk loci in *SOS2* that may be associated with IOP and POAG, which could be involved in the signaling pathways and developmental processes that underlie the risk for IOP elevation [113, 114]. Similarly, case GEL-S08-01 with CG and features of autosomal recessive Knobloch syndrome (KS) had homozygous missense variants in *COL18A1*. KS is typically associated with high myopia, retinal detachment, lens subluxation, and occipital encephalocele [131]. Other ocular anomalies, such as early-onset and acute angle closure glaucomas, have also been observed in KS patients [132, 133]. To the best of our knowledge, only three studies in literature have reported *COL18A1*-associated angle closure glaucomas in KS patients, suggesting a role for *COL18A1* in iridocorneal angle closure [112, 132, 133].

Less than half of the CG cohort in the GE100KGP (44%; 34/78) had PCG, while 56% (44/78) of the families had secondary glaucoma. Furthermore, of the secondary group, 55% (24/44) had glaucomas associated with non-acquired ocular anomalies whereas 45% (20/44) had non-acquired systemic diseases (Fig. 1A). Patients with syndromic features requires a multidisciplinary team approach and input by specialist paediatricians to reduce co-morbidities. The large majority of families (74%; 58/78) remain unsolved (Fig. 1C), and thus require further investigation for novel genes or regulatory elements, and implementation of improved tools to facilitate the diagnosis. Furthermore, acquired conditions, that are not present at birth, may also be responsible for the CG in a proportion of the unexplained cases, including traumas, steroid-induced glaucoma, uveitis, tumours, retinopathy of prematurity, and surgeries [2, 134–136].

Comprehensive diagnostic assessment capturing all relevant clinical information is of utmost importance, as the primary diagnosis in the clinic can impact the selection of gene panel and subsequent results. In family GEL-007, the proband and her sister were found to harbour a likely pathogenic homozygous missense variant in *SLC4A11* (c.1343G>A p.(Gly448Asp)), a gene known to be associated with autosomal recessive congenital hereditary endothelial dystrophy (CHED). Glaucoma is a known associate feature of CHED [137–139], and can lead to misdiagnosis due to overlap in clinical features during early childhood [140]. However, in the case of this family, the patients were not available for clinical re-examination to confirm the correct diagnosis. Thorough evaluation should be carried out when glaucoma is suspected to ensure accurate disease phenotyping, and applying a mixed gene panel (including anterior segment, cornea, cataract and glaucoma genes) can increase the molecular diagnosis rate. Similarly, a 27-year-old male patient GEL-064-01 presented with multisystemic features including CMT disease, demyelinating polyneuropathy, severe autism, depression, anxiety, had mobility issues, and hydromyelia. He was found to harbour two compound heterozygous pathogenic variants in the *SBF2* gene (c.620G>T p.(Gly207Val) and c.2536+1G>A), both found at the boundaries of exons 7 and 20, respectively. Variants in *SBF2* are known to be associated with Charcot-Marie-Tooth disease type 4B2 accompanied by early-onset glaucoma (CMT4B2, MIM: 604563) [141, 142]. Establishing the clinical diagnosis positively influenced our analysis and made it possible to draw a genetic conclusion. CG families who have a molecularly-confirmed diagnosis enable genotype-phenotype correlations to be established and future prognostic indication.

Miscommunication of clinical phenotype between clinicians and clinical scientists can lead to diagnostic errors, such as failing to order the necessary test, or making errors during analysis and variant interpretation due to phenotypic terminologies being missing, imprecise, or misinterpreted [143]. For example, clinicians may apply terms to describe the patient’s current phenotype without consideration of modifying factors such as surgery i.e. a presenting adult with PCG, who may have had several surgeries and now displays corectopia or develops cataracts or corneal decompensation, as opposed to the naïve (pre-intervention) congenital disease features. Providing more information on the patient’s medical presentation would aid the interpretation process and boost the probability of diagnosing the patient [143]. The proper use of HPO terminology will eliminate misinterpretations during the analysis, thereby improving diagnostics [143].

In this cohort, 8 unrelated patients had 8 unique VUSs, which require further investigation. Interestingly, only 1 of the VUS cases was a trio (i.e. patient with both parents) while the remaining were duos (a patient with a single parent) or singletons (patient only). A recent study by Rehm, et al. (2023) demonstrated that the use of trios reduced the rates of inconclusive results compared to the use of less-than-trio (18.9% versus 27.6%, respectively) [144]. With the increased adoption of high-throughput NGS technologies, approximately 40% of total variants are inconclusive and are considered VUS [145]. Nonetheless, variant interpretation has been improved by standardised classification (such as the use of the ACMG guidelines), establishment of publicly available databases integrating supporting evidence from epidemiological, clinical, structural, and functional data (such as gnomAD [103], ClinVar [109], TopMed [104], etc.), and collaborative efforts of researchers and expert working groups to systematically curate the variant data and review protocols of variant interpretation [146–148], as well as the utilisation of *in silico* tools that have the potential to enhance pathogenicity predictions of new variants [105, 149, 150].

## Conclusion

Understanding the aetiology of CG is crucial to delivering the most suitable clinical management and genetic counselling, though it remains challenging given the variable penetrance and genetic heterogeneity associated with the disease. The majority of the CG cases are still genetically undiagnosed possibly due to complex causative factors such as gene modifiers, non-coding variants, novel genes, variants of unknown significance, or non-Mendelian aetiologies (such as epigenetic markers), or non-genetic causes that are not considered in the current analysis pipelines. Combining various technologies such as long-read sequencing, RNA sequencing, and multiomics with regular re-analysis of the genomic data could help resolve these limitations. In addition, sequencing additional family members and conducting segregation analysis can help exclude many non-pathogenic variations. Herein, we demonstrate that through systematic analysis of gene panels, we were able to effectively improve the proportion of genetically diagnosed CG families within the GE100KGP cohort to 26%. By expanding the genetic spectrum of CG, our knowledge of the underlying biological pathways may ultimately help personalise the treatment of glaucoma.

### Electronic supplementary material

Below is the link to the electronic supplementary material.

Supplementary Material 1

Supplementary Material 2

## Data Availability

Source code available from: https://github.com/omayma-alsaei/gel-pcg.
