# Supplementary material for: Increasing the diagnostic yield of childhood glaucoma cases recruited into the 100,000 Genomes Project
Source: BMC Genomics. 2024 May 16;25:484. doi: 10.1186/s12864-024-10353-8 (PMC11097485; doi:10.1186/s12864-024-10353-8)
Supplement: Supplementary file 2 — Supplementary Material 2 [file 12864_2024_10353_MOESM2_ESM.pdf]

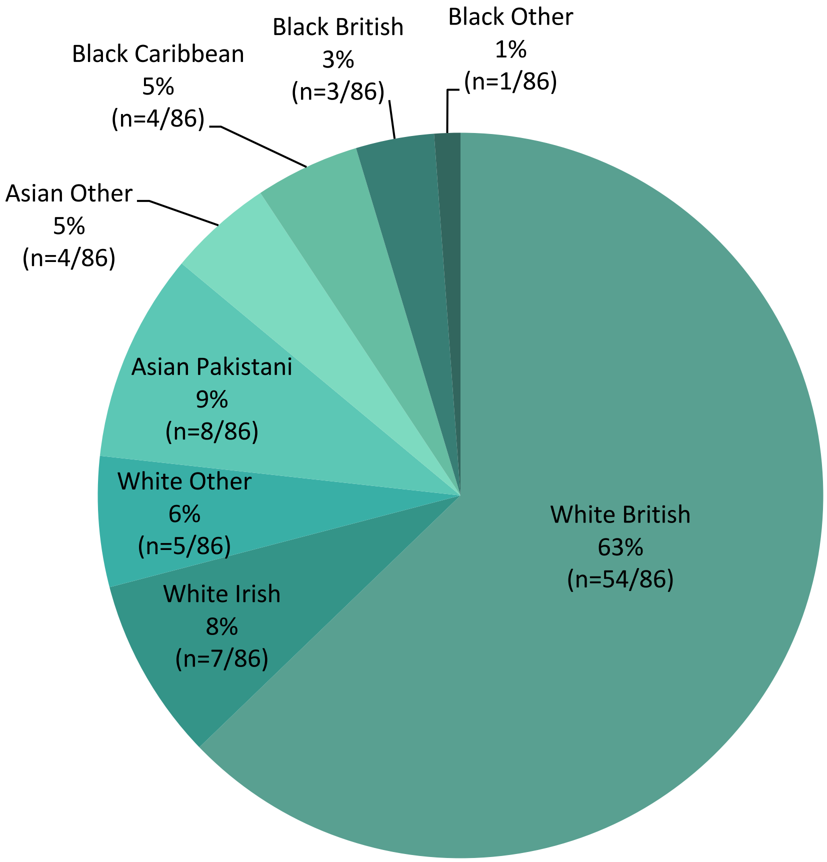

Figure S1. Demographics and diagnostic rate of childhood glaucoma (CG) cohort in the Genomics England 100,000 Genomes Project. Distribution of participants according to their ethnic backgrounds, with statistically higher male percentage distributed among the different ethnic groups compared to females (*p-value* < 0.05). Average sex ratio is 5 males to 3 females per ethnic group.
